# Intergenerational Transmission of Obesity from Mothers to Their Offspring: Trends and Associated Factors Derived from the Malaysian National Health and Morbidity Survey (NHMS)

**DOI:** 10.3390/nu14112186

**Published:** 2022-05-24

**Authors:** Nur Nadia Mohamed, Abdul Jalil Rohana, Noor Aman A Hamid, Frank B. Hu, Vasanti S. Malik, Muhammad Fadhli Mohd Yusoff, Tahir Aris, The Global Nutrition and Epidemiologic Transition Initiative (GNET)

**Affiliations:** 1 Department of Community Medicine, School of Medical Sciences, Universiti Sains Malaysia, Kubang Kerian 16150, Malaysia; annenad2301@gmail.com (N.N.M.); na.hamid@usm.my (N.A.A.H.); 2 The Global Nutrition and Epidemiologic Transition Initiative (GNET), Department of Epidemiology, Harvard T.H. Chan School of Public Health, Boston, MA 02118, USA; fhu@hsph.harvard.edu (F.B.H.); vmalik@hsph.harvard.edu (V.S.M.); 3 Department of Nutrition, Harvard T.H. Chan School of Public Health, Boston, MA 02115, USA; 4 Department of Nutritional Sciences, Faculty of Medicine, University of Toronto, Toronto, ON M5S 1A1, Canada; 5 Institute for Public Health, Ministry of Health Malaysia, Blok B5 & B6, Kompleks NIH, No.1, Jalan Setia Murni U13/52, Seksyen U13, Bandar Setia Alam, Shah Alam 40170, Malaysia; fadhli_my@moh.gov.my; 6 Institute of Medical Research, Ministry of Health Malaysia, Jalan Pahang, Kuala Lumpur 50588, Malaysia; tahir.a@moh.gov.my

**Keywords:** intergenerational obesity, overweight, mother–child pairs, Malaysia

## Abstract

Along with the increasing overweight and obesity trends among adults and children globally, numerous studies have suggested a strong association between maternal overweight and obesity among their offspring. We sought to report the prevalence and associated factors of intergenerational overweight and obesity among mother–child pairs in Malaysia from 2006 to 2015. Data were analysed from three waves of the Malaysian National Health and Morbidity Survey, a population-based cross-sectional study conducted in 2006, 2011 and 2015. A mother and the youngest child from each household formed ‘mother–child pairs’ and were grouped according to their body mass index categories. A multivariable logistic regression model was performed to determine the factors associated with overweight mother/overweight child pairs (OWM/OWC), with normal weight mother/normal weight child pairs (NWM/NWC) as the reference group. The prevalence of OWM/OWC increased from 15.3% to 21.7%, while the prevalence of NWM/NWC decreased from 28.4% to 23.8% between 2006 and 2015. Older maternal age and having primary and secondary education levels were positively associated with OWM/OWC. Conversely, older child age, Chinese ethnicity, large household size and low-income households were inversely associated with OWM/OWC. In conclusion, intergenerational weight gain is a worrisome trend in Malaysia. These findings may help in guiding priority setting for obesity prevention strategies in Malaysia.

## 1. Introduction

The epidemic of overweight and obesity has become one of the most significant challenges to public health globally. It has been estimated that 120 million disability-adjusted life-years and 4 million of all of the deaths in the population worldwide were attributed to overweight and obesity [1]. Recent findings reported that there was a 50% to 80% increase in the prevalence of overweight and obesity across the globe over the past 35 years, with a higher prevalence in women [2]. The number of overweight and obese children has also risen from 4% to 18% over the past four decades [3].

Overweight or obesity has been observed among both adults and children living in the same household, and this trend is apparent particularly among parents and children. A strong relationship between maternal obesity and obesity among their offspring has been reported in numerous studies [4,5,6,7,8]. The intergenerational overweight and obesity can occur either through the shared environment or genetic inheritance [4,9,10]. There are a few theories that support the intergenerational overweight and obesity, such as the theory of Developmental Origins of Health and Disease, which posits that early-life exposure to the maternal environment may promote the development of chronic diseases through foetal programming [11]. On the other hand, the Maternal Resources Hypothesis suggests that childhood obesity is the consequence of socioeconomic evolution over the past century, such as a decrease in maternal physical activity and improved nutrition [12]. As a result, maternal energy resources, both body mass and adiposity, are accumulated together with a reduction in maternal metabolic function. The offspring of a mother with obesity will be born with impaired metabolic function, which is linked to obesity [13]. The undesirable intergenerational cycle of obesity may take place because the process of intergenerational overweight and obesity starts in early life and continues through childhood. Children with obesity are prone to developing overweight or obesity during adulthood, which is linked to a higher risk of cardiometabolic diseases [14,15].

Evaluating the intergenerational overweight and obesity, specifically related to mothers and offspring, is critical, as this would be an important point of intervention for future prevention of cardiometabolic diseases for mothers and their children. Until recently, there have been a limited number of studies that have reported the prevalence of overweight among mothers and offspring living in the same household [16,17,18,19,20,21,22,23,24], including Malaysia. Most of these studies were conducted in low- and middle-income countries, except for one study that included data from 12 countries of different income levels [16]. Based on the existing literature, the prevalence was from 0.6% in the Gaza Strip to 40.0% in Mexico [20,21].

In Malaysia, only a single study has reported the prevalence of overweight among mother–child pairs [18]. This study was conducted in Segamat, a small district located in the southern part of the country; however, the samples did not include the entire Malaysian population. Besides, no study to date has evaluated trends in the prevalence of overweight mother–child pairs. Additionally, there is limited literature on the factors associated with overweight and obesity in mother–child pairs [21]. Therefore, in this study, we aimed to determine the prevalence and associated factors of overweight and obesity among mother–child pairs using data from three waves of a nationally representative survey in Malaysia from 2006 to 2015.

## 2. Materials and Methods

### 2.1. Study Design

The Malaysian National Health and Morbidity Survey (NHMS) is a repeated cross-sectional survey. Initially, it was a ten-yearly survey. It was first conducted in 1986 in Peninsular Malaysia. The second and third surveys were carried out in Sabah and Sarawak in 1996 and 2006. The survey became a five-yearly survey after the NHMS 2006 [25]. It is a household-based survey to obtain information on health status, health needs and expenditures of the Malaysian population. Each respective survey was conducted in a different household. The survey applied a two-stage stratified random sampling scheme, consisting of Enumeration Blocks (EB) and Living Quarters (LQ). Detailed procedures of data collection for the NHMS have been described previously [25,26,27].

### 2.2. Ethical Approval

Ethical approval for this study was granted by the Medical Research Ethics Committee (MREC), the Ministry of Health Malaysia (NMRR-17-2714-38075) and the Human Research Ethics Committee of Universiti Sains Malaysia (USM/JEPeM/17110579).

### 2.3. Study Sample

Generally, Malaysia consists of two main regions: Peninsular Malaysia and East Malaysia. East Malaysia is comprised of the states Sabah and Sarawak. Data from the year 1986 survey were not included because it was only conducted in Peninsular Malaysia. The second survey was conducted in the year 1996 including Sabah and Sarawak. Even so, there was a ten-year gap between the second and the third survey, which was conducted in 2006 due to the financial constraints for conducting the survey. In 2010, the Minister of Health Malaysia started to recommend and allocate specific budgets for annual surveys to provide updated information for policymakers. Hence, the following surveys were carried out every five years. During this study, the Director General of Health Malaysia granted us approval and permission to analyse the data in 2018.

Data were pooled from three waves of the NHMS, from 2006, 2011 and 2015. All extracted data were re-identified and anonymised. In order to match the mother–child pairs, ‘mother’ and ‘child’ were determined manually based on their relationship to the head of the household, sex and age. In Malaysian culture, men are usually acknowledged as the head of the household. Hence, for a woman who was married to the head of the household, she would be recorded as ‘mother’. However, in certain single-headed families, the head of the household is a woman if a single mother is responsible for decision-making in the household. Households were excluded if: (1) there was no information on mother or child available in the household, (2) the person living in the household was living alone or with friends, (3) the youngest child in the family was older than 17 years, (4) there was a single father living in the household, (5) the relationship to the head of the household could not be determined or (6) individuals with incomplete data on height and weight.

A child was defined as a person aged less than 18 years [28]. In this study, only the youngest child between 5 to 17 years in the household was selected and paired with their mother to be included. If there were more than one child aged 5 to 17 years in the household, the youngest was chosen, similarly to the previous mother–child pair studies [29,30,31]. The youngest child of the family was selected because the previous studies have reported that they were at a higher risk of being overweight or obese compared to the oldest and middle child [32,33,34]. Moreover, it has been suggested that the parents tend to be more indulgent when feeding their youngest child than the oldest child [35]. A previous study has demonstrated that an indulgent parenting style was significantly associated with a higher BMI of the children [36]. Most of the household studies used the youngest child in the family due to their vulnerabilities toward food insecurity, which is detrimental to their nutritional status. The children less than five years of age were not included because of the differences in the definition of underweight, overweight and obesity in this age group [37].

The majority of the households were excluded because the child data were not available for each household. The other reasons for exclusion are listed in Table A1. Finally, a total of 6005 mother–child pairs were obtained for the year 2006, while 2957 and 2871 mother–child pairs were acquired for the years 2011 and 2015, respectively.

### 2.4. Body Mass Index (BMI) of the Participants

As part of the NHMS purposes, the body weight of the mothers and their child were measured using a Tanita Personal Scale HD 319, while height was measured using a SECA 206 Body Meter. All tools were validated and calibrated prior to data collection [25,26,27]. Body mass index (BMI) was calculated using the index of height and weight (weight in kilogram/(height in meter)^2^). The classification of maternal BMI was based on the World Health Organisation [38]. Mothers were grouped into three BMI categories: underweight (BMI less than 18.5 kg/m^2^), normal weight (BMI between 18.5 and 24.9 kg/m^2^) and overweight (BMI of 25 kg/m^2^ and above). The BMI of children was categorised based on the World Health Organisation Growth Reference, using BMI-for-age z-scores [39]. The children were categorised into underweight (BMI z-score < –2SD), normal weight (BMI z-score between –2SD and +1SD) and overweight (BMI z-score > +1SD).

As the national survey was not explicitly designed to evaluate the intergenerational transmission of weight status from mother to child, maternal BMI data were matched to offspring BMI data to create a mother–child pair variable as an outcome of the study. Ultimately, nine mother–child BMI categories were created: (1) underweight mother/underweight child (UWM/UWC), (2) underweight mother/normal weight child (UWM/NWC), (3) underweight mother/overweight child (UWM/OWC), (4) normal weight mother/underweight child (NWM/UWC), (5) normal weight mother/normal weight child (NWM/NWC), (6) normal weight mother/overweight child (NWM/OWC), (7) overweight mother/underweight child (OWM/UWC), (8) overweight mother/normal weight child (OWM/NWC) and (9) overweight mother/overweight child (OWM/OWC).

### 2.5. Statistical Analyses

Data were analysed using SPSS, version 24 (IBM, Chicago, IL, USA). All sociodemographic information was analysed as categorical variables and presented as frequencies (*n*) and percentage (%). The continuous variables such as age, BMI and household size were reported as mean and standard deviation (SD). One-way ANOVA was performed to compare means of maternal and child BMI across three survey years. Post hoc analysis using Dunnett’s C procedure was selected because the variances were unequal. It was conducted to identify specific pairs of survey years showing significant differences in means of maternal and child BMI.

Household size was divided into three groups: small (less than five persons in the household), medium (five to seven persons in the household) and large (more than seven persons in the household) [40]. Ethnicity was categorised into four groups based on the predominant ethnic groups in the country: Malay, Chinese, Indian and Other. Maternal education level was divided into four categories: no education, primary education, secondary education and tertiary education [41]. Household income was categorised into five quintiles for each survey, where Quintile 1 represented 20% of the lowest household income (the most impoverished household), and Quintile 5 indicated the top 20% of the highest household income (the most affluent household). Detailed household income ranges for each quintile group for each survey are described in Table A2.

Family structure was divided into single-parent or dual-parent households. Single-parent refers to the household with a single-mother, while dual-parent refers to a household with both a father and a mother. The residential area of the participants was categorised into urban (area with at least 10,000 people living in the area) and rural (less than 10,000 people living in the appointed area) by the Department of Statistics of Malaysia [25,26].

Simple and multivariable logistic regression models were conducted to determine the factors associated with OWM/OWC with NWM/NWC as the reference group [21]. The independent variables included in the analysis were maternal age, child age, child sex, household size, ethnicity, parental education level, household income, family structure and residential area. The selection of the potential variables was based on the previous study which investigated factors associated with nutritional status of mother–child dyads [21]. All the variables with a *p*-value < 0.25 in the simple logistic regression analysis were included in the multivariable logistic regression models [42,43,44]. Multicollinearity and interaction terms were checked. Multicollinearity was checked with the variance inflation factor (VIF). Values of VIF of more than 10 indicate that multicollinearity exists between the independent variables. The interaction terms were checked by testing possible two-way interactions of the independent variables, such as maternal education level with household income and maternal age with maternal education level. Possible interactions were checked based on the literature and expert opinion. In this study, potential interactions between maternal education level with household income were checked. Based on the literature, we postulated that the association between education level and OWM/OWC would be different across household income based on the previous studies [45,46]. We also examined the potential interactions between maternal age and maternal education level. Again, based on the previous evidence, we postulated that the association between maternal education and OWM/OWC would be varied by their age. Previous studies have reported that there was a significant association between age and education level [47,48]. A previous study also demonstrated that the frequency of low education was higher in the oldest group, while high education levels predominated in the youngest group [49]. Model fitness was tested using a classification table and the Hosmer–Lemeshow goodness-of-fit test [42]. The findings were reported as adjusted odds ratios (AOR) with 95% confidence intervals (CI), and *p*-values. Findings with a *p*-value < 0.05 were considered statistically significant.

## 3. Results

### 3.1. Characteristics of Mother–Child Pairs

Table 1 presents the sociodemographic characteristics of the mother–child pairs in this study by survey year. For each survey year, the majority of mothers were 41 to 50 years of age (2006 = 41.5%, 2011 = 42.2%, 2015 = 40.8%), Malay (2006 = 60.9%, 2011 = 62.3%, 2015 = 66.7%), had completed a secondary education level (2006 = 54.3%, 2011 = 56.6%, 2015 = 56.4%), living in a medium household size (2006 = 50.5%, 2011 = 54.0%, 2015 = 48.6%), had a monthly household income within the 5th quintile category (2006 = 24.5%, 2011 = 22.5%, 2015 = 23.6%), had a dual-parent family (2006 = 94.7%, 201 = 94.8%, 2015 = 93.0%) and resided in an urban area (2006 = 56.5%, 2011 = 57.2%, 2015 = 57.1%). Among children, the majority were aged 5 to 9 years (2006 = 59.7%, 2011 = 55.1%, 2015 = 55.8%), with an equal proportion of boys and girls across each survey year.

The BMI characteristics of mother–child pairs are shown in Table 2. Among the mothers, there was a significant difference in mean maternal BMI across survey years (*p* < 0.001). Findings from the post hoc analysis revealed that the mean of maternal BMI in 2015 (27.36 kg/m^2^, SD = 5.55) was significantly higher than the mean of maternal BMI in 2006 (26.19 kg/m^2^, SD 5.19, *p* < 0.001). The prevalence of underweight mothers was 4.1% in 2006, 3.4% in 2011 and 2.8% in 2015. Among normal weight mothers, the prevalence was 40.6% in 2006, 38.2% in 2011 and 33.8% in 2015. In 2006, the prevalence of overweight mothers was 34.3%, while in 2011 and 2015, the prevalence was 35.2% and 34.9%, respectively. The prevalence of mothers with obesity was 21.0% in 2006, 23.2% in 2011 and 28.5% in 2015.

Similar to the mother, the mean of child BMI was significantly different across the survey years (*p* < 0.001). The mean of child BMI in 2015 (18.28 kg/m^2^, SD = 5.05) was significantly greater than the mean BMI in 2006 (17.26 kg/m^2^, SD = 4.45, *p* < 0.001). In this study, we found that the prevalence of underweight children was 10.2% in 2006, 10.1% in 2011 and 7.6% in 2015. Besides that, the prevalence of normal weight children for the years 2006, 2011 and 2015 was 67.2%, 66.3% and 64.1%, respectively. Among overweight children, the prevalence was 12.9% in 2006, 12.4% in 2011 and 13.0% in 2015. In contrast, the prevalence of children with obesity was 9.7%, 11.2% and 15.3% in 2006, 2011 and 2015.

### 3.2. The Prevalence of Different BMI Categories by Mother–Child Pair

**Figure 1:**
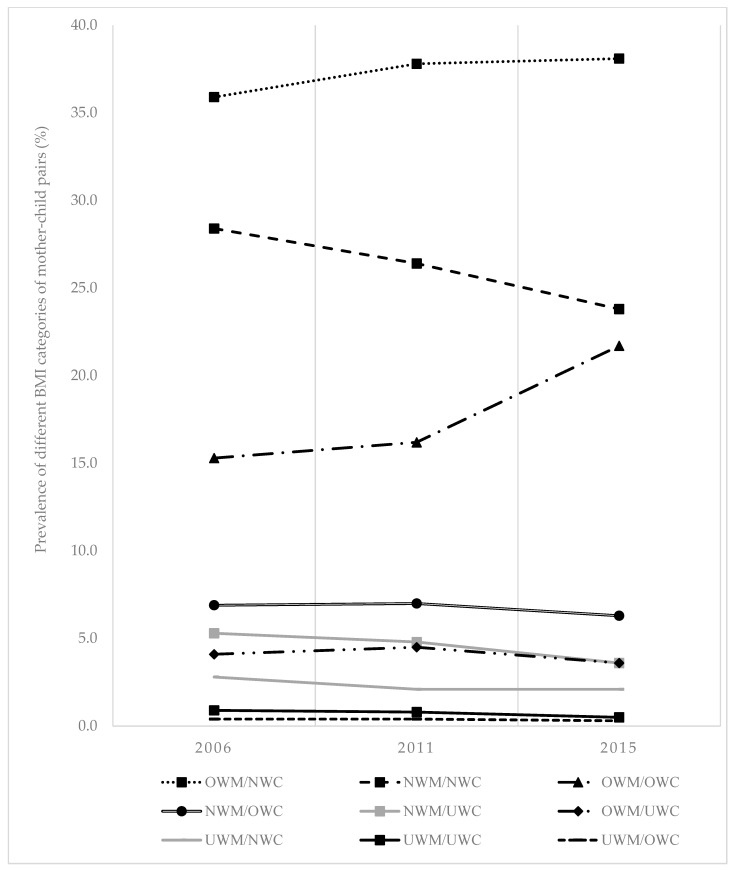
Prevalence of different BMI categories of mother–child pairs (UWM/UWC = underweight mother/underweight child; UWM/NWC = underweight mother/normal weight child; UWM/OWC = underweight mother/overweight child; NWM/UWC = normal weight mother/underweight child; NWM/NWC = normal weight mother/normal weight child; NWM/OWC = normal weight mother/overweight child; OWM/UWC = overweight mother/underweight child; OWM/NWC = overweight mother/normal weight child; OWM/OWC = overweight mother/overweight child).

Figure 1 and Table 3 show the trend of prevalence of different BMI categories of mother–child pairs across the three survey years. The majority of mother–child pairs across all survey years were categorised as OWM/NWC. The prevalence of OWM/NWC was 35.9% in 2006, 37.8% in 2011 and 38.1% in 2015. In contrast, the prevalence of NWM/NWC decreased from 28.4% to 23.8% over the period. There was a marked increase in OWM/OWC from 15.3% in 2006, 16.2% in 2011 and 21.7% in 2015. The other BMI categories for mother–child pairs (NWM/OWC, NWM/UWC, OWM/UWC, UWM/NWC, UWM/UWC and UWM/OWC) showed a decrease in prevalence.

### 3.3. Factors Associated with Overweight Mother/Overweight Child Pair (OWM/OWC)

Table 4 presents the findings of a simple logistic regression analysis for the factors associated with OWM/OWC in Malaysia for the years 2006, 2011 and 2015. In 2006, maternal age greater than 50 years (OR = 3.21, 95% CI = 2.12–4.87, *p* < 0.001), child age between 10 and 14 years (OR = 2.13, 95% CI = 1.78–2.55, *p* < 0.001), Indian (OR = 1.39, 95% CI = 1.03–1.88, *p* = 0.031) and primary education level of the mother (OR = 1.77, 95% CI = 1.26–2.50, *p* = 0.001) were positively associated with OWM/OWC. However, large household size (OR = 0.64, 95% CI = 0.47–0.87, *p* = 0.005), Chinese ethnicity (OR = 0.43, 95% CI = 0.34–0.55, *p* < 0.001) and Quintile 1 of household income (OR = 0.53, 95% CI = 0.39–0.72, *p* < 0.001) were inversely associated with OWM/OWC.

In the 2011 survey, maternal age more than 50 years old (OR = 2.07, 95% CI = 1.15–3.74, *p* = 0.015), child age between 10 and 14 years old (OR = 1.96, 95% CI = 1.52–2.53, *p* < 0.001) and no education (OR = 1.74, 95% CI = 1.01–2.99, *p* = 0.044) were positively associated with OWM/OWC. Chinese ethnicity had a 62% less probability to become OWM/OWC in comparison to Malay (OR = 0.38, 95% CI = 0.27–0.53, *p* < 0.001).

In addition, in the 2015 survey, maternal age above 50 years (OR = 2.01, 95% CI = 1.20–3.38, *p* = 0.008), child age between 10 and 14 years (OR = 1.47, 95% CI= 1.15–1.88, *p* = 0.002) and primary education level of the mother (OR = 1.62, 95% CI = 1.15–2.29, *p* = 0.006) showed positive association with OWM/OWC. Meanwhile, large household size (OR = 0.52, 95% CI = 0.31–0.87, *p* = 0.013), Chinese ethnicity (OR = 0.43, 95% CI = 0.31–0.61, *p* < 0.001) and living in an urban area (OR = 0.77, 95% CI = 0.62–0.96, *p* = 0.019) were less likely to be associated with OWM/OWC.

Findings from the multivariable logistic regression model of the factors associated with OWM/OWC in Malaysia for the years 2006, 2011 and 2015 are shown in Table 5. For the 2006 survey, maternal age greater than 50 years (AOR = 2.87, 95% CI = 1.77–4.66, *p* < 0.001), child age between 10 and 14 years (AOR = 1.59, 95% CI = 1.30–1.96, *p* < 0.001) and primary education level of the mother (AOR = 2.24, 95% CI = 1.49–3.36, *p* < 0.001) were positively associated with OWM/OWC. In contrast, large household size (AOR = 0.62, 95% CI = 0.44–0.86, *p* = 0.005), Chinese ethnicity (AOR = 0.33, 95% CI = 0.25–0.43, *p* < 0.001) and Quintile 1 of household income (AOR = 0.45, 95% CI = 0.31–0.64, *p* < 0.001) were inversely associated with OWM/OWC.

In the 2011 survey, only child age between 10 and 14 years old (AOR = 1.76, 95% CI = 1.30–2.36, *p* < 0.001) was positively associated with OWM/OWC. In addition, we found an inverse association between large household size (AOR = 0.56, 95% CI = 0.34–0.91, *p* = 0.019) and Chinese ethnicity (AOR = 0.30, 95% CI = 0.21–0.43, *p* < 0.001) with OWM/OWC.

In 2015, maternal age over 50 years (AOR = 2.11, 95% CI = 1.16–3.84, *p* = 0.015) and primary education level of the mother (AOR = 2.00, 95% CI = 1.32–3.03, *p* = 0.001) were positively associated with OWM/OWC. In contrast, child age between 15 and 17 years (AOR = 0.60, 95% CI = 0.40–0.89, *p* = 0.012), large household size (AOR = 0.44, 95% CI = 0.25–0.76, *p* = 0.003), Chinese ethnicity (AOR = 0.44, 95% CI = 0.31–0.63, *p* < 0.001) and Quintile 2 of household income (AOR = 0.61, 95% CI = 0.41–0.92, *p* = 0.017) were inversely associated with OWM/OWC.

## 4. Discussion

Based on our analysis of a national sample from Malaysia, the prevalence of intergenerational overweight and obesity from mother to offspring increased over ten years, while the prevalence of normal weight mother–child pairs decreased.

In low- and middle-income countries (LMICs), the prevalence of overweight and obesity has increased rapidly over a relatively short period [50,51]. These trends have been driven in part by rapid economic growth and urbanisation in many LMICs, which have resulted in nutrition and lifestyle transitions linked to weight gain [52,53,54,55]. With increasing incomes, food and lifestyle habits have changed as people were able to obtain more income and purchasing power to buy various types of food [56]. Consequently, traditional diets which were high in vegetables and whole grains have been replaced with foods high in refined grains, added sugar, animal products and saturated fats [57].

Dietary changes from the consumption of whole foods to high intake of processed foods that are high in calories but lack nutrients have been observed in many countries, including Malaysia [58]. Malaysia has also been undergoing a nutrition transition towards a high intake of fat, sugar and animal products since the 1960s [59]. Food availability of calories from animal products and total sugars in Malaysia has increased over four decades [60]. The accessibility of high-calorie food with a low nutrient quality combined with a low level of physical activity owing to economic development and globalisation has accelerated the rate of obesity in Malaysia [61], which may in part explain the rise in maternal–child overweight that we observed in this study.

In the year 2006, the prevalence of OWM/OWC in this study was 15.3%, which was lower than the overweight mother–child pairs reported in Croatia (25.3%) over the same period [24]. A decade later, the prevalence of OWM/OWC in the 2015 survey (21.7%) was found to be higher than the findings reported in other developing countries, such as Colombia (12.4%), China (13.4%), Samoa (16.9%) and India (20.7%) [16,17]. However, the prevalence of OWM/OWC found in our study was lower in comparison to that reported in South Africa (22.4%), Brazil (28.4%) and Mexico (40.0%) [16,21].

In this study, the association of intergenerational overweight and obesity was positively associated with maternal age. This finding might be due to young mothers who were more physically active and conscious of their health than older mothers. Besides, as adults age, they tend to gain weight despite having usual dietary intakes and behaviour because of lower energy requirements, reductions in physical activity and a lower metabolic rate [62].

In contrast, an inverse association of intergenerational overweight and obesity was observed among younger children. Mothers also have a crucial role in shaping dietary intake habits in their children. It has been shown that dietary resemblance between parents and children was stronger among young children [63]. As children grow, it has been hypothesised that they become more aware and influenced by their body image, and they may participate in more recreational physical activities in comparison to younger children [64]. In our finding, children aged between 15 and 17 years were at high risk of intergenerational overweight and obesity in the 2011 survey. However, this association was not significant. In the 2015 survey, the children aged between 15 and 17 had a lower risk of intergenerational overweight and obesity. It is difficult to explain these findings, and hence, further studies are needed to identify the factors that lead to such differences.

In this study, large household size and low household income were inversely associated with intergenerational obesity, consistent with the findings in another mother–child pair study [21]. As reported earlier, the household income can influence dietary intake and behaviours of household members [65]. The increase of income is the main contributor to the rise in food availability, which has been related to a higher incidence of obesity [66]. Low-income households may have challenges in purchasing food due to higher costs. In contrast, individuals from high-income households can spend more money on various types of foods [67]. It is possible that OWM/OWC was less likely to occur in large or low-income households due to limited income available to purchase food.

Our study also found that Chinese ethnicity was inversely associated with intergenerational overweight and obesity. This finding is consistent with previous studies that have reported Chinese ethnicity to be inversely associated with the risk of overweight and obesity compared to other ethnicities [68,69,70]. It is possible that the difference could be due in part to the different ethnic-dietary basis. The Chinese population in Malaysia were found to have healthy dietary patterns with higher daily intakes of fruits and vegetables compared to other ethnicities [71,72]. In another study, a greater number of Chinese children had achieved the recommended vegetable intake compared to Malay children [73].

Our study also found that the increased risk of intergenerational overweight and obesity was associated with mothers who had a lower education level. Mothers with a lower education level may have a lack of knowledge on healthy eating and lifestyle. In contrast, mothers with higher education levels tend to place more attention on healthy dietary behaviours, particularly vegetable and fruit intakes [74]. It has also been shown that women with higher education levels had better diet quality as compared to their counterparts [75].

The strategies to prevent intergenerational overweight and obesity could be more efficient when focusing on the specific group. Based on the findings from this study, preventive measures of intergenerational overweight and obesity could be targeted to older mothers, children aged between 10 and 14 years and mothers with primary and secondary education levels.

The main strength of this study is the use of repeated measurements of nationally representative data, which allowed us to examine the trend of intergenerational overweight and obesity in Malaysia across ten years. However, several limitations should be acknowledged. The data utilised in this study were cross-sectional data. Hence, we were not able to infer causality. We used repeated cross-sectional data, and as such, the change in BMI category for each mother–child pair could not be assessed because different mother–child pairs were included in each cross-sectional assessment. Besides that, the data were not adjusted for other obesity risk factors such as physical activity, dietary quality and smoking status. Potential residual confounding may have influenced the findings of this study.

## 5. Conclusions

In conclusion, there is a worrying trend in the prevalence of intergenerational overweight and obesity in Malaysia. Until recently, one in five households in Malaysia had an overweight mother and an overweight child. The determinants of intergenerational overweight and obesity were older maternal age and lower education level. Older children, large household size, low household income and Chinese ethnicity were inversely associated with overweight mother–child pairs. Given that the prevalence of overweight and obese mother–child pairs is on the rise, intervention programmes for weight management should be implemented at the household level among mothers and their children.

## Figures and Tables

**Table 1:** Sociodemographic characteristics of mother–child pairs in the National Health and Morbidity Survey of Malaysia in 2006, 2011 and 2015.

Characteristics of Mother–Child Pairs	2006		2011		2015	
	*n*	%	*n*	%	*n*	%
Maternal age in years, mean (SD)	40.9 (7.5)		41.5 (7.3)		41.7 (7.7)	
<30	529	8.8	187	6.3	206	7.2
31–40	2335	38.9	1175	39.7	1106	38.5
41–50	2490	41.5	1248	42.2	1170	40.8
51 and above	651	10.8	347	11.7	389	13.5
Total	6005		2957		2871	
Ethnicity						
Malays	3659	60.9	1842	62.3	1915	66.7
Chinese	926	15.4	443	15.0	331	11.5
Indian	485	8.1	212	7.2	194	6.8
Others	935	15.6	460	15.6	431	15.0
Total	6005		2957		2871	
Maternal education level						
Tertiary	401	6.7	510	17.4	614	21.6
Secondary	3246	54.3	1662	56.6	1606	56.4
Primary	1832	30.7	609	20.7	527	18.5
None	497	8.3	156	5.3	101	3.5
Total	5976		2937		2848	
Household size, mean (SD)	5.16 (1.82)		5.23 (1.72)		4.87 (1.62)	
Small (<5 persons)	2393	39.9	1091	37.0	1319	45.9
Medium (5–7 persons)	3034	50.5	1594	54.0	1394	48.6
Large (>7 persons)	577	9.6	266	9.0	158	5.5
Total	6004		2951		2871	
Household income						
Quintile 5	1429	24.5	665	22.5	677	23.6
Quintile 4	1396	23.9	633	21.4	607	21.1
Quintile 3	960	16.4	536	18.1	635	22.1
Quintile 2	1310	22.4	559	18.9	461	16.1
Quintile 1	747	12.8	432	14.6	491	17.1
Total	5842		2825		2871	
Family structure						
Dual-parent family	5682	94.7	2803	94.8	2671	93.0
Single-parent family	319	5.3	154	5.2	200	7.0
Total	6001		2957		2871	
Residential area						
Rural	2613	43.5	1265	42.8	1233	42.9
Urban	3392	56.5	1692	57.2	1638	57.1
Total	6005		2957		2871	
Child age in years, mean (SD)	9.2 (3.6)		9.7 (3.7)		9.6 (3.7)	
5–9	3587	59.7	1629	55.1	1603	55.8
10–14	1730	28.8	892	30.2	876	30.5
15–17	688	11.5	436	14.7	392	13.7
Total	6005		2957		2871	
Sex of child						
Girls	2906	48.4	1464	49.5	1405	48.9
Boys	3099	51.6	1493	50.5	1466	51.1
Total	6005		2957		2871	

Note: SD = standard deviation.

**Table 2:** Body mass index characteristics of mother–child pairs in the National Health and Morbidity Survey of Malaysia in 2006, 2011 and 2015.

Characteristics of Mother–Child Pairs	2006		2011		2015		*p*-Value ^a^
	(*n* = 6005)		(*n* = 2957)		(*n* = 2871)		
	*n*	%	*n*	%	*n*	%	
Maternal BMI, mean (SD)	26.19 (5.19)		26.66 (5.39)		27.36 (5.55)		<0.001 ^b^
Underweight	248	4.1	100	3.4	81	2.8	
Normal weight	2438	40.6	1130	38.2	970	33.8	
Overweight	2060	34.3	1041	35.2	1003	34.9	
Obese	1259	21.0	686	23.2	817	28.5	
Child BMI, mean (SD)	17.26 (4.45)		17.78 (4.66)		18.28 (5.05)		<0.001 ^b^
Underweight	617	10.2	299	10.1	219	7.6	
Normal weight	4034	67.2	1961	66.3	1839	64.1	
Overweight	774	12.9	367	12.4	374	13.0	
Obese	580	9.7	330	11.2	439	15.3	

Note: BMI = body mass index; SD = standard deviation. ^a^ One-way ANOVA test. ᵇ Post hoc analysis: 2015 vs. 2011 (*p* < 0.001), 2015 vs. 2006 (*p* < 0.001), 2011 vs. 2006 (*p* < 0.001).

**Table 3:** Prevalence of mother–child pairs by BMI categories for years 2006, 2011 and 2015.

Mother–Child Pairs Categories	2006		2011		2015	
	(*n* = 6005)		(*n* = 2957)		(*n* = 2871)	
	*n*	%	*n*	%	*n*	%
UWM/UWC	52	0.9	25	0.8	13	0.5
UWM/NWC	171	2.8	62	2.1	60	2.1
UWM/OWC	25	0.4	13	0.4	8	0.3
NWM/UWC	320	5.3	142	4.8	104	3.6
NWM/NWC	1705	28.4	782	26.4	684	23.8
NWM/OWC	413	6.9	206	7.0	182	6.3
OWM/UWC	245	4.1	132	4.5	102	3.6
OWM/NWC	2158	35.9	1117	37.8	1095	38.1
OWM/OWC	916	15.3	478	16.2	623	21.7

Note: UWM/UWC = underweight mother/underweight child; UWM/NWC = underweight mother/normal weight child; UWM/OWC = underweight mother/overweight child; NWM/UWC = normal weight mother/underweight child; NWM/NWC = normal weight mother/normal weight child; NWM/OWC = normal weight mother/overweight child; OWM/UWC = overweight mother/underweight child; OWM/NWC = overweight mother/normal weight child; OWM/OWC = overweight mother/overweight child.

**Table 4:** Simple logistic regression model for the factors associated with overweight mother/overweight child pairs in Malaysia for 2006, 2011 and 2015.

Risk Factors	2006 (*n* = 2621)				2011 (*n* = 1260)				2015 (*n* = 1307)			
	OR	95% CI		*p*-Value	OR	95% CI		*p*-Value	OR	95% CI		*p*-Value
Maternal age												
≤30	1.00				1.00				1.00			
31–40	1.74	1.21, 2.49		0.003	1.19	0.71, 2.01		0.510	1.51	0.96, 2.38		0.078
41–50	3.22	2.25, 4.60		<0.001	1.66	0.99, 2.78		0.054	1.72	1.09, 2.70		0.019
51 and above	3.21	2.12, 4.87		<0.001	2.07	1.15, 3.74		0.015	2.01	1.20, 3.38		0.008
Child age												
5–9	1.00				1.00				1.00			
10–14	2.13	1.78, 2.55		<0.001	1.96	1.52, 2.53		<0.001	1.47	1.15, 1.88		0.002
15–17	1.44	1.11, 1.87		0.006	1.25	0.89, 1.76		0.193	0.83	0.60, 1.13		0.235
Child sex												
Girl	1.00				1.00				1.00			
Boy	0.90	0.76, 1.05		0.180	1.13	0.90, 1.42		0.294	1.08	0.87, 1.35		0.465
Household size ^a^												
Small	1.00				1.00				1.00			
Medium	0.83	0.70, 0.99		0.033	0.80	0.63, 1.01		0.062	0.99	0.79, 1.24		0.923
Large	0.64	0.47, 0.87		0.005	0.64	0.41, 1.01		0.055	0.52	0.31, 0.87		0.013
Ethnicity												
Malay	1.00				1.00				1.00			
Chinese	0.43	0.34, 0.55		<0.001	0.38	0.27, 0.53		<0.001	0.43	0.31, 0.61		<0.001
Indian	1.39	1.03, 1.88		0.031	1.44	0.93, 2.22		0.100	1.23	0.79, 1.92		0.359
Other	0.53	0.41, 0.67		<0.001	0.56	0.39, 0.79		0.001	0.79	0.58, 1.07		0.122
Maternal education level												
Tertiary	1.00				1.00				1.00			
Secondary	1.49	1.07, 2.08		0.018	1.29	0.95, 1.74		0.100	1.60	1.21, 2.12		0.001
Primary	1.77	1.26, 2.50		0.001	1.20	0.83, 1.74		0.330	1.62	1.15, 2.29		0.006
None	0.84	0.53, 1.31		0.438	1.74	1.01, 2.99		0.044	1.66	0.91, 3.02		0.097
Household income ᵇ												
Quintile 5	1.00				1.00				1.00			
Quintile 4	1.24	0.99, 1.56		0.064	1.08	0.78, 1.51		0.637	1.01	0.73, 1.40		0.932
Quintile 3	1.23	0.96, 1.58		0.107	1.19	0.84, 1.69		0.328	1.34	0.98, 1.84		0.067
Quintile 2	0.82	0.64, 1.04		0.094	1.09	0.76, 1.55		0.649	0.92	0.65, 1.31		0.649
Quintile 1	0.53	0.39, 0.72		<0.001	1.14	0.78, 1.67		0.510	1.22	0.87, 1.72		0.247
Family structure												
Dual-parent family	1.00				1.00				1.00			
Single-parent family	0.88	0.61, 1.26		0.474	1.41	0.84, 2.37		0.191	1.08	0.69, 1.67		0.739
Residential area												
Rural	1.00				1.00				1.00			
Urban	1.12	0.95, 1.32		0.184	1.00	0.80, 1.27		0.971	0.77	0.62, 0.96		0.019

Note: CI = confidence interval; OR = odds ratio. Statistically significant (*p*-value < 0.05) are highlighted in bold. ^a^ Small = less than five persons; medium = five to seven persons; large = more than seven persons in the household. ᵇ Quintile 5 is the most affluent household while Quintile 1 is the most impoverished household.

**Table 5:** Multivariable logistic regression analysis for the factors associated with overweight mother/overweight child pairs in Malaysia for 2006, 2011 and 2015.

Risk Factors	2006 (*n* = 2621)			2011 (*n* = 1260)			2015 (*n* = 1307)		
	AOR	95% CI	*p*-Value	AOR	95% CI	*p*-Value	AOR	95% CI	*p*-Value
Maternal age									
≤30	1.00				1.00				1.00
31–40	1.72	1.17, 2.53	0.005	0.94	0.54, 1.65	0.836	1.47	0.92, 2.37	0.108
41–50	2.82	1.91, 4.18	<0.001	1.14	0.64, 2.02	0.657	1.68	1.03, 2.74	0.038
51 and above	2.87	1.77, 4.66	<0.001	1.27	0.64, 2.54	0.497	2.11	1.16, 3.84	0.015
Child age									
5–9	1.00				1.00				1.00
10–14	1.59	1.30, 1.96	<0.001	1.76	1.30, 2.36	<0.001	1.21	0.91, 1.61	0.185
15–17	0.92	0.67, 1.26	0.594	1.02	0.67, 1.55	0.937	0.60	0.40, 0.89	0.012
Household size ^a^									
Small	1.00				1.00				1.00
Medium	0.87	0.72, 1.04	0.132	0.82	0.63, 1.07	0.150	0.93	0.73, 1.19	0.573
Large	0.62	0.44, 0.86	0.005	0.56	0.34, 0.91	0.019	0.44	0.25, 0.76	0.003
Ethnicity									
Malay	1.00				1.00				1.00
Chinese	0.33	0.25, 0.43	<0.001	0.30	0.21, 0.43	<0.001	0.44	0.31, 0.63	<0.001
Indian	1.31	0.95, 1.82	0.101	1.29	0.82, 2.04	0.273	1.31	0.83, 2.07	0.254
Other	0.69	0.53, 0.89	0.005	0.57	0.39, 0.84	0.005	0.78	0.56, 1.09	0.142
Maternal education level									
Tertiary	1.00				1.00				1.00
Secondary	1.74	1.20, 2.52	0.003	1.26	0.88, 1.79	0.209	1.73	1.26, 2.38	0.001
Primary	2.24	1.49, 3.36	<0.001	1.21	0.76, 1.92	0.423	2.00	1.32, 3.03	0.001
None	1.15	0.67, 1.95	0.615	1.65	0.84, 3.23	0.147	1.93	1.00, 3.73	0.052
Household income ᵇ									
Quintile 5	1.00				1.00				1.00
Quintile 4	1.05	0.81, 1.35	0.726	1.01	0.71, 1.45	0.960	0.76	0.54, 1.10	0.149
Quintile 3	1.06	0.80, 1.40	0.684	0.99	0.66, 1.47	0.954	0.90	0.63, 1.29	0.577
Quintile 2	0.67	0.50, 0.89	0.005	0.86	0.57, 1.29	0.471	0.61	0.41, 0.92	0.017
Quintile 1	0.45	0.31, 0.64	<0.001	0.83	0.52, 1.32	0.434	0.81	0.55, 1.21	0.309

Note: CI = confidence interval; AOR = adjusted odds ratio. Statistically significant (*p*-value < 0.05) are highlighted in bold. No multicollinearity and interaction were detected. Hosmer–Lemeshow test (2006: *p* = 0.961; 2011: *p* = 0.944; 2015: *p* = 0.602). ^a^ Small = less than five persons; medium = five to seven persons; large = more than seven persons in the household. ᵇ Quintile 5 is the most affluent household while Quintile 1 is the most impoverished household.

## Data Availability

The National Health and Morbidity Survey data analysed in this study are not publicly available due to privacy and ethical issues. The data can be requested from the Institute of Public Health, Medical Research Ethics Committee (MREC), and the Ministry of Health Malaysia.

## Appendix A

**Table A1:** Exclusion criteria of the households.

Description	2006	2011	2015
Total number of households (N)	15,316	7638	8427
Reasons for exclusion			
No data on child in the household	3797	1893	2063
Living alone	2144	1015	1325
Child’s age above 17 years old in the household	1373	890	1062
Living with friends	207	167	142
Relationship to the head of the household cannot be determined	1216	441	992
Missing data on height and weight	574	275	258
Mother–child pairs obtained	6005	2957	2871

## Appendix B

**Table A2:** Household income range for every quintile.

Quintile Income	Household Income Range (RM)		
	2006	2011	2015
Quintile 1 (lowest income)	<500	<950	<1200
Quintile 2	500–949	950–1727	1200–1999
Quintile 3	950–1499	1728–2799	2000–3181
Quintile 4	1500–2499	2800–4599	3182–5299
Quintile 5 (highest income)	2500 and above	4600 and above	5300 and above

Note: RM = Ringgit Malaysia (1 USD = RM 4.22).
